# Buckling Analysis of Thin-Walled Laminated Plates Considering In-Plane and Out-of-Plane Coupling Effects Under Complex In-Plane Loads

**DOI:** 10.3390/ma19153297

**Published:** 2026-08-03

**Authors:** Zbigniew Kolakowski, Andrzej Teter

**Affiliations:** 1 Department of Strength of Materials (K12), Lodz University of Technology (TUL), Stefanowskiego 1/15, 90-924 Lodz, Poland; zbigniew.kolakowski@p.lodz.pl; 2 Department of Applied Mechanics, Faculty of Mechanical Engineering, Lublin University of Technology (LUT), Nadbystrzycka 36, 20-618 Lublin, Poland

**Keywords:** coupling stiffness, complex in-plane load, general laminate

## Abstract

**Highlights:**

- The coupling B-submatrix has an effect on the buckling behaviour of thin-walled laminated plates.
- The inclusion of the coupling B-submatrix leads to decreased bifurcation loads.
- Coupling effects represented by the B-submatrix result in stiffness reduction coefficients within the beam model.

**Abstract:**

This study investigates the buckling behaviour of thin-walled rectangular laminated plates under complex in-plane loading. Linear variations in the normal transverse load components and parabolic variations in the shear load components are assumed on the plate edges. The in-plane load distributions reproduce the design solutions reported in the literature. The starting point for the considerations in this study is the classical laminated plate theory (CLPT). It focuses on cases when the laminate coupling stiffness B-submatrix components are non-zero. Six cases of in-plane loading in the pre-buckling stage are investigated. Ten examples of plates made of general laminates with different stacking sequences and the same thickness are tested. The effect of selected components of the coupling B-submatrix on the stability of the rectangular laminated plates is determined. Eigen-problem solutions for the laminated plates are obtained under complex in-plane loading, demonstrating that the proper assessment of eigen-values must include a detailed analysis of all stiffness matrix components and reduction coefficients in beam modelling. For all cases investigated in this study, the inclusion of the B-submatrix decreases the bifurcation loads.

## 1. Introduction

Plate structures are widely used in modern lightweight load-bearing structures owing to their adaptability to complex design requirements. Depending on their fixture (i.e., boundary conditions), these structures can exhibit, e.g., low torsional stiffness. Assessing the stability of such structures poses a significant challenge, particularly with advanced composite materials.

The literature on this subject generally lacks studies investigating the impact of complex in-plane loading on laminated plate structures. Structures made of general laminates with different ply stacking sequences are particularly interesting [1]. Laminates of this type can be used for practical applications owing to their thermal stability and resistance to warping during production. Their use enables optimised mechanical properties for a given structure, depending on the application.

One study [2] investigated the post-buckling behaviour of composite plates under combined in-plane shear, compression, and lateral loading using an Element-Based Lagrangian Formulation. In [3], the buckling behaviour of laminated composite plates reinforced with carbon nanotubes (CNTs) and carbon fibres under in-plane loading was analysed by developing a soft computing framework that coupled isogeometric analysis (IGA) with Murakami’s zigzag. Murakami’s zigzag Function (MZZF) is a kinematic enrichment used in the mechanics of laminated composite and sandwich materials [4]. A review paper [5] synthesised the use of analytical, FEM-based, and other computational methods, along with existing experimental findings, in the analysis of the buckling behaviour of laminated panels under compression and shear. Another study [6] examined the buckling and post-buckling behaviour of a rectangular angle-ply plate under biaxial loading. It was found that the impact of coupling conditions on the post-buckling structural stiffness caused mainly a change in the buckling mode shape. A new method for solving the deflection problem in rectangular composite plates under uniform in-plane (biaxial compression and shear) and transverse loads was proposed in [7]. One study [8] investigated the buckling of laminated composite plates with higher-order theory general lamination configurations. Another study [9] proposed a simple, higher-order theory-based in-plane displacement model for the buckling analysis of laminated composite plates with general lamination configurations. A three-dimensional buckling analysis of functionally gradient material (FGM) plates under non-uniform in-plane compression was undertaken in [10]. One study [11] proposed a method for predicting the buckling loads of composite plates based on a buckling analysis of individual loads and provided a corresponding prediction formula. The buckling of rectangular isotropic plates under patch and concentrated loads for different boundary conditions was investigated in [12]. Another paper [13] presented the approximate buckling interaction curves for anisotropic plates, showing high correlation between the approximate method and FEM results. The post-buckling behaviour of composite plates under combined shear, compression, and transverse loads was investigated in [14]. One study [15] presented the impact of different parameters, including stacking sequence, number of plies, load, and boundary conditions, on the buckling and post-buckling behaviour of composite plates under bidirectional compression, shear, and transverse loads.

Additional modes of deformation can affect buckling behaviour, reduce the buckling load, or change the post-buckling stiffness. The most effective tool for their analysis is to use FEM shell models. Nonetheless, the modelling of thin-walled load-bearing structures entails high CPU (Central Processing Unit) costs and, at the same time, loss of solution generality. Consequently, research must be conducted to develop design methods that optimise the mechanical properties of such structures with minimal time expenditure. For the design of load-bearing structures made of multiple laminates with different stacking sequences, one must have in-depth knowledge of their behaviour under complex mechanical and thermal loads. The thermal stability of stacking sequences in laminates is indispensable for the fabrication of these structures by autoclaving, as laminates are cured at elevated temperatures.

**Figure 1:**
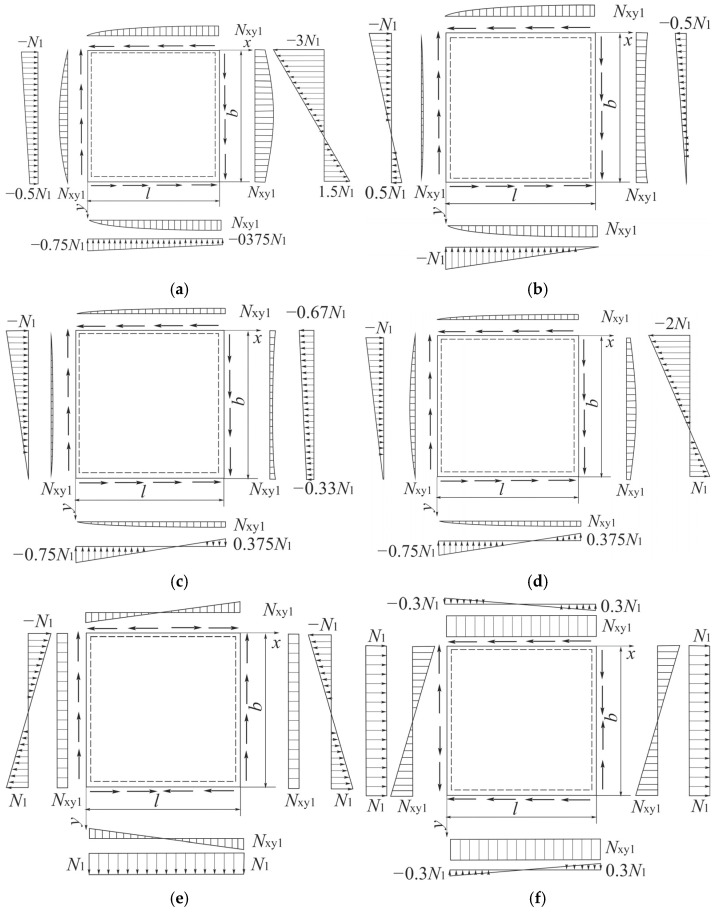
Tested cases of in-plane loading applied to the plates in the pre-buckling stage: (**a**) Case 1, (**b**) Case 2, (**c**) Case 3, (**d**) Case 4, (**e**) Case 5, and (**f**) Case 6.

In this study, all types of ABD stiffness matrix coupling are examined, with a special focus on the B-submatrix. This study uses the classical laminated plate theory (CLPT) [16,17,18,19]. Six cases of in-plane loading applied to the edges of rectangular laminated plates in the pre-buckling stage are tested (Figure 1). The cases presented correspond to the load distributions known from the literature [20,21] arising from the practical application of plate elements. Ten examples of ply stacking sequences in thermostable laminates are investigated.

## 2. Formulation of the Problem

The classical laminated plate theory (CLPT) [16,17,18,19] assumes that composite materials used for fabricating the plates are subject to Hooke’s law. Key equations in CLPT derived from the variational approach are presented in brief in Appendix A.

In this study, the plates are subjected to complex in-plane loading. It was assumed that the normal loads *N*_x_ and *N*_y_ would vary linearly in the 0*x*- and 0*y*-axis directions. In contrast, the shear components *N*_xy_ would vary parabolically (Figure 1). The distributions shown in Figure 1 have been reported in the literature [16,17,18,22,23,24] for isotropic materials. The distributions of shear stresses are described by the relationship presented by Zhuravskii, which is a well-known formula in the literature [24,25,26].

The transverse load distributions (Figure 1) on the laminate plate edges can be calculated from the following relationships:

(1) $$N_{x}=C_{3}+C_{6}\frac{x}{b}+C_{7}\frac{y}{b}+2C_{9}\frac{xy}{b^{2}},\;N_{y}=C_{1}+C_{4}\frac{x}{l}+C_{5}\frac{y}{l}+2C_{8}\frac{xy}{l^{2}}.\;N_{xy}=-C_{2}-C_{5}\frac{x}{l}-C_{6}\frac{y}{b}-C_{8}\frac{x^{2}}{l^{2}}-C_{9}\frac{y^{2}}{b^{2}}$$

where *C*_1_–*C*_9_ denote unknown coefficients, and the plate dimensions are denoted by *l* and *b* (see Figure 1).

The unknown coefficients *C*_1_–*C*_9_ are calculated from the condition that they take the set values at characteristic points on the plate edges (Figure 1). It can be observed that eight constants in the plate corners predetermine the linear transverse loads *N*_x_ and *N*_y_ (these are the constants *C*_1_, *C*_3_–*C*_9_). It must be noted that such load distributions (Equation (1)) satisfy the identity conditions (Equation (A9)) of the first two equilibrium equations for the plate element at pre-buckling for all the tested load cases (Figure 1). This means that the static equations are satisfied. Moreover, the assumption of the distributions of the normal loads *N*_x_ and *N*_y_ in the four corners yields different coefficients *C*_1_–*C*_9_. The shear load *N*_xy_ also changes. The pre-buckling transverse load components (Equation (1)) do not depend on the stiffness matrix.

Moreover, in accordance with Equations (A6) and (A1), on the edges *x =* 0; *L* and *y =* 0; b, the transverse loads (Equation (1)) correspond to the edge loads: $N_{x}^{0},N_{y}^{0},N_{xy}^{0}$.

From the differential equilibrium equations (Equation (A9)), after considering Equations (A10) and (A1)–(A4), what follows is a system of the following three linear differential equations of equilibrium expressed by the displacements *u*, *v*, *w*:

(2) $$\begin{array}{c} A_{11}\frac{\partial^{2}u}{\partial x^{2}}+2A_{16}\frac{\partial^{2}u}{\partial x\partial y}+A_{66}\frac{\partial^{2}u}{\partial y^{2}}+A_{16}\frac{\partial^{2}v}{\partial x^{2}}+\left(A_{12}+A_{66}\right)\frac{\partial^{2}v}{\partial x\partial y}+A_{26}\frac{\partial^{2}v}{\partial y^{2}}-B_{11}\frac{\partial^{3}w}{\partial x^{3}}-\\ 3B_{16}\frac{\partial^{3}w}{\partial x^{2}\partial y}-\left(B_{12}+2B_{66}\right)\frac{\partial^{3}w}{\partial x\partial y^{2}}-B_{26}\frac{\partial^{3}w}{\partial y^{3}}=0,\\ A_{16}\frac{\partial^{2}u}{\partial x^{2}}+(A_{12}+A_{66})\frac{\partial^{2}u}{\partial x\partial y}+A_{26}\frac{\partial^{2}u}{\partial y^{2}}+A_{66}\frac{\partial^{2}v}{\partial x^{2}}+2A_{26}\frac{\partial^{2}v}{\partial x\partial y}+A_{22}\frac{\partial^{2}v}{\partial y^{2}}-B_{16}\frac{\partial^{3}w}{\partial x^{3}}-(B_{12}+\\ 2B_{66})\frac{\partial^{3}w}{\partial x^{2}\partial y}-3B_{26}\frac{\partial^{3}w}{\partial x\partial y^{2}}-B_{22}\frac{\partial^{3}w}{\partial y^{3}}=0,\\ D_{11}\frac{\partial^{4}w}{\partial x^{4}}+4D_{16}\frac{\partial^{4}w}{\partial x^{3}\partial y}+2\left(D_{12}+2D_{66}\right)\frac{\partial^{4}w}{\partial x^{2}\partial y^{2}}+4D_{26}\frac{\partial^{4}w}{\partial x\partial y^{3}}+D_{22}\frac{\partial^{4}w}{\partial y^{4}}-B_{11}\frac{\partial^{3}u}{\partial x^{3}}-\\ 3B_{16}\frac{\partial^{3}u}{\partial x^{2}\partial y}-\left(B_{12}+2B_{66}\right)\frac{\partial^{3}u}{\partial x\partial y^{2}}-B_{26}\frac{\partial^{3}u}{\partial y^{3}}-B_{16}\frac{\partial^{3}v}{\partial x^{3}}-\left(B_{12}+2B_{66}\right)\frac{\partial^{3}v}{\partial x^{2}\partial y}-3B_{26}\frac{\partial^{3}v}{\partial x\partial y^{2}}-\\ B_{22}\frac{\partial^{3}v}{\partial y^{3}}-N_{x}\frac{\partial^{2}w}{\partial x^{2}}-2N_{xy}\frac{\partial^{2}w}{\partial x\partial y}-N_{y}\frac{\partial^{2}w}{\partial x^{2}}=0. \end{array}$$

No general solution exists for this system of equations. We know of only two solutions for special cases of ply layups, namely for regular antisymmetric cross-ply laminated plates and for regular antisymmetric angle-ply laminated plates [17,18]. The above system can only be solved approximately.

## 3. Results

To determine the minimum bifurcation loads for rectangular composite plates under complex loading, the following system of differential equations (Equation (2)) was solved. The solution of the system of differential equations in Equation (2) is expressed as follows:

(3) $$u=\sum_{n=1}^{N}\sum_{m=1}^{M}U_{mn}\;cos\alpha x\;sin\beta y,\;v=\sum_{n=1}^{N}\sum_{m=1}^{M}V_{mn}\;sin\alpha x\;cos\beta y,\;w=\sum_{n=1}^{N}\sum_{m=1}^{M}W_{mn}\;sin\alpha x\;sin\beta y,$$

where

(4) $$\alpha =\frac{m\pi }{l},\;\;\;\;\;\;\beta =\frac{n\pi }{b}.$$

Every single constituent in the series (Equation (3)) corresponds to a general solution for regular antisymmetric cross-ply laminated plates under uniform compression in the 0*x*-axis direction. Also, in [21], similar trigonometric functions were assumed in a longitudinal direction. After substituting the predicted solutions of Equation (3) into the system of equations (Equation (2)), the linear problem of stability was solved using the Bubnov–Galerkin method [20,21,22,24,26,27,28,29]. This method is based on the condition of orthogonality between two functionals, namely the functional of the equilibrium equation and the functional of the solution in the area *S = l × b*. Listed in the Appendix, the equations derived by variation methods define the orthogonality of these functionals. From Equation (A5), after considering Equations (A10) and (3), we ultimately obtain a system of three equations:

(5) $$\int_{S}\left[N_{x,x}+N_{xy,y}\right]cos(\frac{i\pi x}{l})sin(\frac{j\pi y}{b})dS=0,\;\int_{S}\left[N_{xy,x}+N_{y,y}\right]sin(\frac{i\pi x}{l})cos(\frac{j\pi y}{b})dS=0,\;\int_{S}\left[M_{x,x}+2M_{xy,xy}+M_{y,y}+N_{x}w_{,x}+{2N}_{x}w_{,xy}+N_{y}w_{,y}\right]sin(\frac{i\pi x}{l})sin(\frac{j\pi y}{b})dS=0,$$

for *i =* 1, *I = M*; for *j =* 1, *J = N.*

The algebraic system of linear equations in Equation (5) creates a square matrix with dimensions (3*M*N) × (3*M*N). By setting the determinant for this equation system to zero, we obtain the lowest bifurcation loads (denoted as *N*_1_). The corresponding lowest buckling stress was determined using the following formula:

(6) $$\sigma_{cr}=N_{1}/t,$$

where the denotation *N*_1_ is derived in accordance with Figure 1. According to Equation (3), the constitutive equations (Equation (A3)) can be written as follows:

(7) $$\begin{array}{c} N_{x}=\sum_{n=1}^{N}\sum_{m=1}^{M}\left[-\alpha A_{11}U_{mn}-\beta A_{12}V_{mn}+\left(B_{11}\alpha^{2}+B_{12}\beta^{2}\right)W_{mn}\right]sin\alpha x\;sin\beta y+\\ \sum_{n=1}^{N}\sum_{m=1}^{M}\left[A_{16}\left(\beta U_{mn}+\alpha V_{mn}\right)-2\alpha \beta B_{16}W_{mn}\right]cos\alpha xcos\beta y,\\ N_{y}=\sum_{n=1}^{N}\sum_{m=1}^{M}\left[-\alpha A_{12}U_{mn}-\beta A_{22}V_{mn}+\left(B_{12}\alpha^{2}+B_{22}\beta^{2}\right)W_{mn}\right]sin\alpha x\;sin\beta y+\\ \sum_{n=1}^{N}\sum_{m=1}^{M}\left[A_{26}\left(\beta U_{mn}+\alpha V_{mn}\right)-2\alpha \beta B_{26}W_{mn}\right]cos\alpha xcos\beta y,\\ N_{xy}=\sum_{n=1}^{N}\sum_{m=1}^{M}\left[-\alpha A_{16}U_{mn}-\beta A_{26}V_{mn}+\left(B_{16}\alpha^{2}+B_{26}\beta^{2}\right)W_{mn}\right]sin\alpha x\;sin\beta y+\\ \sum_{n=1}^{N}\sum_{m=1}^{M}\left[A_{66}\left(\beta U_{mn}+\alpha V_{mn}\right)-2\alpha \beta B_{66}W_{mn}\right]cos\alpha xcos\beta y,\\ M_{x}=\sum_{n=1}^{N}\sum_{m=1}^{M}\left[-\alpha B_{11}U_{mn}-\beta B_{12}V_{mn}+\left(D_{11}\alpha^{2}+D_{12}\beta^{2}\right)W_{mn}\right]sin\alpha x\;sin\beta y+\\ \sum_{n=1}^{N}\sum_{m=1}^{M}\left[B_{16}\left(\beta U_{mn}+\alpha V_{mn}\right)-2\alpha \beta D_{16}W_{mn}\right]cos\alpha xcos\beta y,\\ M_{y}=\sum_{n=1}^{N}\sum_{m=1}^{M}\left[-\alpha B_{12}U_{mn}-\beta B_{22}V_{mn}+\left(D_{12}\alpha^{2}+D_{22}\beta^{2}\right)W_{mn}\right]sin\alpha x\;sin\beta y+\\ \sum_{n=1}^{N}\sum_{m=1}^{M}\left[B_{26}\left(\beta U_{mn}+\alpha V_{mn}\right)-2\alpha \beta D_{26}W_{mn}\right]cos\alpha xcos\beta y,\\ M_{xy}=\sum_{n=1}^{N}\sum_{m=1}^{M}\left[-\alpha B_{16}U_{mn}-\beta B_{26}V_{mn}+\left(D_{16}\alpha^{2}+D_{26}\beta^{2}\right)W_{mn}\right]sin\alpha x\;sin\beta y+\\ \sum_{n=1}^{N}\sum_{m=1}^{M}\left[B_{66}\left(\beta U_{mn}+\alpha V_{mn}\right)-2\alpha \beta D_{66}W_{mn}\right]cos\alpha xcos\beta y. \end{array}$$

It can easily be noted that in Equation (7), each of the generalised internal transverse loads is expressed via two components in a double series, where the first one contains the factor sinα*x*sinβ*y*, and the other contains cosα*x*cosβ*y*.

This study assumes that the composite plates are simply supported on their edges. Their boundary conditions are as follows:

(8) $$\begin{array}{c} \mathrm{for}x=0,L:\\ \int_{0}^{b}N_{x}dy=0,\;\;\;\;\;\;v=\;0,\;\;\;\;\;\;w=0,\;\;\;\;\;\;\int_{0}^{b}M_{x}dy=0, \end{array}$$

(9) $$\begin{array}{c} \mathrm{for}y=0;b:\\ \int_{0}^{l}N_{y}dy=0,\;\;\;\;\;\;u=0,\;\;\;\;\;\;w\;=0,\;\;\;\;\;\;\int_{0}^{l}M_{y}dy=0. \end{array}$$

The first and last boundary conditions defined in Equations (8) and (9) denote the weak boundary conditions for the internal loads (Equation (7)) on the plate edges. Weak boundary conditions are commonly used in the FEM. It should be stressed that the first of the constituents multiplied by sinα*x*sinβ*y* strictly meets the zeroing condition for the applicable boundary condition, whereas the other constituent is equal to zero.

The study investigated in detail supported rectangular laminated plates with the following dimensions: width *b =* 300 mm, thickness *t =* 2.16 mm, and lengths *l =* 300 mm and *l =* 450 mm. The mechanical constants of the laminate were as follows: Young’s modulus along the fibre direction of 170 GPa and along the fibre transverse direction of 7.6 GPa, respectively; Poisson’s ratio in-plane of 0.36; and Kirchhoff’s modulus in-plane of 3.52 GPa [26]. Two variants of the laminate were tested: one with 18 plies, each 0.12 mm thick, and the other with 16 plies, each 0.135 mm thick.

According to Figure 1, six cases of complex in-plane loads were tested (denoted by Cases 1–6). Table 1 lists ten examples of general laminates that illustrate the selected in-plane and out-of-plane coupling effects [26]. EX-1 through EX-10 denote the tested stacking sequences of the laminate.

**Figure 2:**
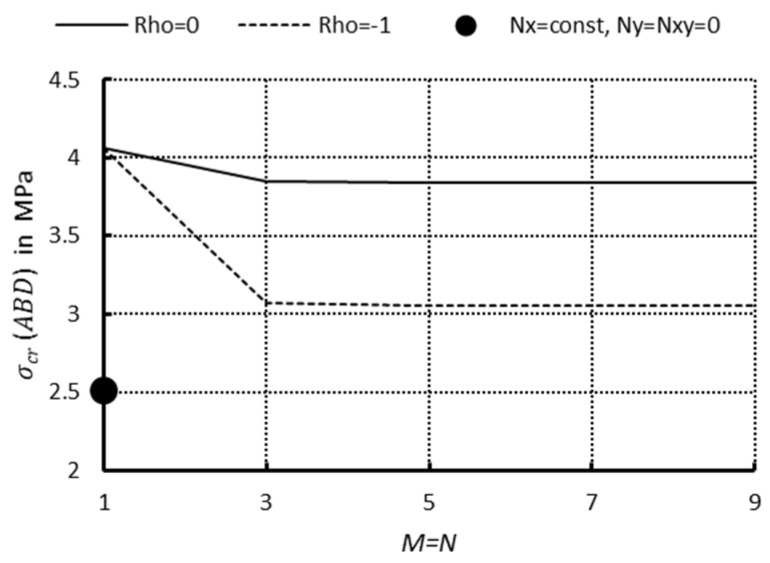
Minimum buckling stress as a function of the number of terms in the series *M = N* (Case 1, EX-3, when *l/b =* 1).

To validate the accuracy of the designed calculation programme, calculations were made for regular antisymmetric cross-ply laminated plates under uniform compression in the 0*x*-axis direction. Detailed calculations were performed for a plate made of the EX-3 laminate with *l*/*b* = 1 and *M = N* = 1. Such a solution is a strict solution of the equation system in Equation (2). According to Equation (6), this yielded the lowest buckling stress of $\sigma_{cr}\;$= $\sigma_{cr}$(ABD) = 2.513MPa, where ABD denotes the case of a laminate with a full stiffness matrix. The dot in Figure 2 represents this value. After that, the convergence of the Galerkin–Bubnov method for the adopted calculation programme was analysed. Detailed calculations were performed for Case 1, laminate EX-3, and *l*/*b* = 1. Figure 2 shows the minimum buckling stress as a function of the number of terms in the series (Equation (3)) when *M = N*.

In the analytical calculations, the authors considered several simplified load cases and several complex ones reported in the literature, obtaining excellent agreement between the results. This was the reason that no FEM verification was carried out in this study.

To determine the constants *C*_1_*–C*_9_ (Equation (1)) for Case 1 in Figure 1, it is sufficient to assign eight conditions at the plate corners; hence, the values of *C*_2_ can be arbitrary. Therefore, the following coefficient was introduced:

(10) $$\rho =\frac{N_{xy1}}{N_{x1}}=\frac{C_{2}}{C_{3}},$$

with its value being ρ = 0 or −1. Figure 2 shows agreement between the two ρ values. Sufficient agreement was already obtained for *M = N* = 3, and later it was assumed that *M = N* = 7. The values of σ_cr_ for both ρ values for *M = N* ≥ 7 change by one digit in the fifth significant place. Convergence was investigated for a number of laminate configurations in order to verify the correctness of the programme’s calculations. For *M = N* = 7, the calculated determinant is a square matrix comprising over 21,000 matrix elements. The accuracy is below 0.001 percent, which the authors consider to be sufficient convergence.

This paper presents the minimum buckling stresses (i.e., bifurcations) obtained for six load cases and ten laminate examples with different stacking sequences for two variants that describe in-plane and out-of-plane effects. Variant I describes the complete coupling B-submatrix, while in Variant II, the B-submatrix is ignored (i.e., [B] = 0). Variant II corresponds to a situation when only the last of the equilibrium equations (Equation (5)) is taken into account. The two variants demonstrate the impact of the complete stiffness matrix compared to omitting the coupling B-submatrix.

**Figure 3:**
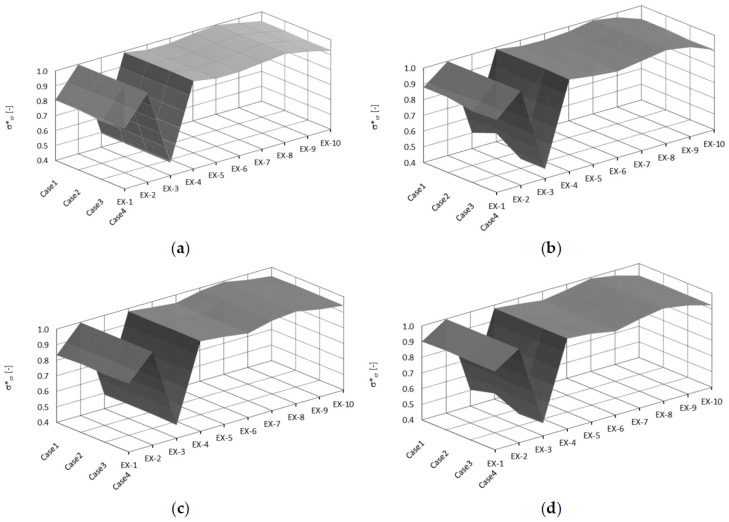
Dimensionless buckling stress in laminated plates (EX-1 through EX-10) under complex in-plane loading for Cases 1–4: (**a**) *l/b =* 1 and ρ = 0; (**b**) *l/b =* 1.5 and ρ = 0; (**c**) *l/b =* 1 and ρ = −1; (**d**) *l/b =* 1.5 and ρ = −1.

Table 2 lists the lowest buckling stresses of all the tested plates subjected to the loads presented in Figure 1a–d (i.e., Cases 1–4), and Table 3 shows the results obtained for Cases 5 and 6 (Figure 1e,f). As can be easily observed in Table 2, the highest values of σ_cr_ (ABD) occur in EX-4 first, followed by EX-2, EX-8, EX-9, EX-7, EX-1, EX-10, EX-6, and EX-5 for two values of *l/b* and ρ. The lowest value of σ_cr_ (ABD) can be observed for EX-3. A comparison of the values of σ_cr_ (ABD) obtained for different loads (Cases 1–4) demonstrates that the highest values were obtained for Case 2, followed by Cases 3–4 and 1. An increase in the *l/b* ratio causes a decrease in the buckling stresses, and the same situation can be observed when the ρ value is changed from 0 to −1. For the discussed load cases (Cases 1–4, Figure 3), the σ_cr_(ABD) values range from over 25 MPa to about 3 MPa.

Numerical calculations for other load cases (Cases 5–6 in Table 2) should also include the shear load *N*_xy_. It was assumed that for Case 5, *N*_xy_ (*x = l*/2, *y =* 0) = 0, while for Case 6, *N*_xy_ (*x = l*/2, *y = b*/2) = 0. For these loads (Cases 5–6), we have nine conditions from which we can unequivocally calculate all coefficients *C*_i_. One cannot, therefore, assign arbitrary values of *ρ*, as can be seen in Case 6 (Figure 1f): the transverse load components are *N*_x_ = *N*_y_ = 0 when *x = l*/2, and *N*_xy_ = 0 when *y = b*/2. The buckling stress is higher for Case 5 than for Case 6. For Cases 5–6, the σ_cr_ values range from approximately 5 MPa to approximately 100 MPa.

After that, the zeroing of the coupling B-submatrix was forced numerically, i.e., [B] = 0 (Variant II). In this case, the system of equations (Equation (5)) was reduced to the last equation. To facilitate the interpretation of the results, dimensionless buckling stresses were introduced and defined as follows:

(11) $$\sigma_{cr}^{*}=\sigma_{cr}(ABD)/\sigma_{cr}(B=0),$$

where σ_cr_(ABD)—minimum buckling stress in Variant I; σ_cr_(B = 0)—minimum buckling stress in Variant II.

**Figure 4:**
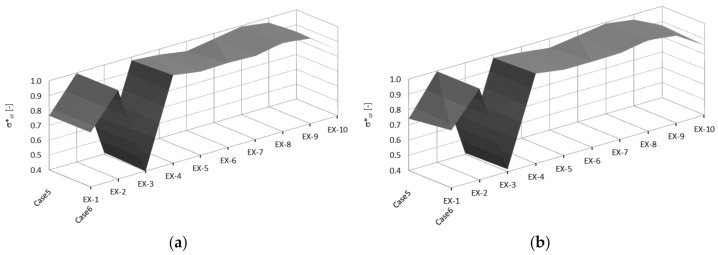
Dimensionless buckling stress in laminated plates (EX-1 through EX-10) under complex in-plane loading for Cases 5–6: (**a**) *l/b =* 1; (**b**) *l/b =* 1.5.

Figure 3 and Figure 4 show the plots of dimensionless buckling stresses $\sigma_{cr}^{*}$ (Equation (11)) for all of the tested examples of plates. The plots demonstrate that including the B-submatrix decreases the lowest buckling stress relative to omitting it. The dimensionless buckling stresses in EX-2 and EX-8 are practically equal to one for the tested load cases (Cases 1–4, Figure 3) and the applied parameters l/b and ρ. The dimensionless buckling stresses of the tested laminates decrease in the order of EX-4, EX-7, EX-6, EX-5, EX-9, and EX-10, and they are not lower than 0.9. The dimensionless buckling stresses of EX-1 do not exceed 0.8, while those of EX-3 decrease even to a value of 0.45.

A similar behaviour pattern can be observed in Figure 4, where the plates were subjected to load Cases 5–6 and two values of the dimensionless length *l/b*. The dimensionless buckling stresses for the laminates EX-2 and EX-8 are practically 1.0, while for the laminates EX-4, EX-5, EX-9, EX-6, and EX-7, these values are greater than 0.9. For EX-10, the dimensionless buckling stress value is above 0.8, whereas for EX-1, it is greater than 0.7. The lowest value, about 0.4, was obtained for EX-3.

A comparison of the dimensionless values in Figure 3 and Figure 4 shows that the lowest values were obtained for the laminate EX-3. For the coupling submatrix, *B*_11_ > 0, *B*_22_ < 0, *B*_11_ = −*B*_22_, while the other submatrix components are equal to zero. The absolute values of *B*_11_ and *B*_22_ are higher than the bending D-submatrix components, making this the only such case the authors know of. The study in [27] introduced the dimensionless reduction coefficients for 1D beam modelling, which were defined as follows:

(12) $$\alpha_{1}=1-\frac{{\left(B_{11}\right)}^{2}}{A_{11}D_{11}},\;\;\;\;\alpha_{2}=1-\frac{{\left(B_{22}\right)}^{2}}{A_{22}D_{22}},\;\;\;\;\alpha_{6}=1-\frac{{\left(B_{66}\right)}^{2}}{A_{66}D_{66}},$$

in the direction of the axis 0*x*, 0*y*, and 0*z*, respectively.

It must be stressed that the ratio $\frac{{\left(B_{ii}\right)}^{2}}{A_{ii}D_{ii}}$ is dimensionless. Table 4 lists the values of the above reduction coefficients in Equation (12) for all the tested laminate configurations, EX-1 through EX-10. For EX-3, the dimensionless reduction coefficients are α_1_ = 0.262, α_2_ = 0.633, and α_6_ = 1 (Table 2). The authors of this paper know of no other case when the reduction coefficient value was below 0.5 for α_1_ or α_2_. Hence, there is a considerable decrease in the buckling stress for Variant I. The second case of the lowest dimensionless stress $\sigma_{cr}^{*}$ was observed for EX-1. The reduction coefficients are α_1_ = 0.837, α_2_ = 0.876, and α_6_ = 0.843 (Table 2). The values of α_1_ and α_2_ are considerably higher than those obtained for EX-3. It should be highlighted that the coupling B-submatrix contains all non-zero components. The *B*_11_ element has the highest absolute value, which is about threefold higher than that of the successive element in the B-submatrix. Moreover, *B*_11_ < 0, and all the other components are non-negative. The third lowest dimensionless stress $\sigma_{cr}^{*}$ was obtained for the EX-10 laminate. In this case, all components of the stiffness and bending submatrices are non-zero. The coefficient values are as follows: α_1_ = 0.885, α_2_ = 0.981, and α_6_ = 0.459 (Table 2). It can be seen that the reduction shear/torsion coefficient value is below 0.5. This probably causes the several-percent reduction in the buckling stress compared to Variant I. In summary, the lowest reduction coefficients for the 1D beam model as defined in Equation (12) are obtained in the following order: EX-3, EX-1, and EX-10. This explains the above-mentioned reduction in buckling stress across all the tested laminates.

The results of the calculations assessing the approximate stability of laminated rectangular plates subjected to complex in-plane loading show that, to assess the impact of submatrix coupling components accurately, one must thoroughly examine all stiffness matrix components and reduction coefficients in a 1D beam model, as defined in Equation (12). It should be noted that the inclusion of the coupling B-submatrix causes the bifurcation load to increase, as is the case when this submatrix is set to zero. The physical interpretation was the attempt to interpret the differences in bifurcation loads, taking into account the values of the dimensionless reduction coefficients for the 1D beam model.

## 4. Conclusions

This paper investigated the stability of rectangular laminated composite plates with a complete stiffness matrix (ABD) under complex in-plane loading. The composite plates were subjected to six complex in-plane loading cases. They were made from laminates with ten different ply stacking sequences, each exhibiting different in-plane and out-of-plane coupling effects. The plates were supported on their edges. The considerations of this study were limited to general thermostable laminates exhibiting all coupling effects. Special focus was put on the non-zero coupling stiffness B-submatrix. The stability problem was solved using the Galerkin–Bubnov method. Two plate lengths were analysed, with the results showing that the lowest buckling stress occurred in the longer plates due to the boundary conditions having a lesser stiffening effect on the plates. An increase in the tangential load components led to higher complex load components, thereby decreasing the buckling stress.

This study investigated the buckling of composite plates with different ply stacking sequences, considering all possible stiffness components (ABD). For eight laminate configurations (EX-2, EX-4-EX-10 in Table 1), the consideration of stiffness matrix coupling caused a slight decrease, up to 10%, in the lowest buckling stresses when compared to the omission of the B-submatrix. For one laminate (EX-1 in Table 1), the bifurcation stress decreased up to 25%, whereas for the laminate EX-3 in Table 1, the decrease was over 50%. A detailed analysis of all stiffness matrix components and reduction coefficients can help predict a significant reduction in the bifurcation load for the complete stiffness matrix compared to case [B] = 0. It is vital to understand the impact of different combinations of in-plane loads on the lowest buckling stresses. Since this aspect requires further research, the authors plan to investigate it in their future studies.

## Figures and Tables

**Table 1:** Analysed cases of columns made of 18-ply and 16-ply laminates [28].

Laminate	Stacking Sequences of the Laminate [26]	Number of Laminate Plies
EX-1	[-45/05/45/-45/0/(45,-45)3/-45/452]	
EX-2	[-45/453/(0,-45)2/0/45/-45/0/45/-45/452/0/-45]	18
EX-3	[9012/06]	
EX-4	[(452,-452)4]	
EX-5	[[(02,902)4]T]	
EX-6	[02,902,454,-454,02,902]	
EX-7	[452,-452,902,02,-452,452,02,902]	16
EX-8	[452,-452,-452,452,-452,452,-452,452]	
EX-9	[452,-452,-302,302,-602,602,-452,452]	
EX-10	[(02,902)2,(-452,452)2]	

**Table 2:** Minimum buckling stress of laminated plates (EX-1 through EX-10, see Table 1) under complex in-plane loading for Cases 1–4 (Variant I—complete stiffness matrix ABD).

Laminate	l/b = 1 and ρ = 0				l/b = 1.5 and ρ = 0			
	Case 1	Case 2	Case 3	Case 4	Case 1	Case 2	Case 3	Case4
EX-1	9.53	19.67	16.95	15.89	7.43	11.85	13.95	13.30
EX-2	12.53	25.86	22.85	20.93	9.37	14.98	18.04	16.80
EX-3	3.84	7.86	6.85	6.44	3.74	6.77	6.20	5.76
EX-4	13.77	28.29	24.80	22.96	10.83	17.33	20.30	18.85
EX-5	7.49	15.43	13.51	12.72	7.06	11.41	12.89	11.98
EX-6	8.24	16.98	14.93	13.96	7.47	12.08	13.68	12.60
EX-7	9.97	20.59	18.50	16.76	8.84	14.40	16.07	14.48
EX-8	12.72	26.38	23.90	21.33	9.83	15.86	19.06	17.09
EX-9	12.18	25.26	22.92	20.44	9.43	15.22	18.32	16.39
EX-10	9.51	19.66	17.60	15.91	7.13	11.49	13.81	12.65
**Laminate**	l/b **= 1 and** ρ **= −1**				l/b **= 1.5 and** ρ **= −1**			
	**Case 1**	**Case 2**	**Case 3**	**Case 4**	**Case 1**	**Case 2**	**Case 3**	**Case 4**
EX-1	7.82	12.74	12.40	11.28	5.79	7.98	9.19	8.52
EX-2	9.75	15.64	15.37	13.88	6.98	9.55	10.98	10.14
EX-3	3.05	4.96	4.82	4.38	2.87	4.29	4.20	3.90
EX-4	10.76	17.31	16.99	15.30	8.07	11.11	12.65	11.59
EX-5	6.01	9.74	9.48	8.71	5.39	7.46	8.39	7.71
EX-6	6.48	10.42	10.20	9.31	5.59	7.71	8.68	7.95
EX-7	7.46	11.89	11.71	10.54	6.34	8.76	9.66	8.80
EX-8	9.30	14.71	14.59	13.03	6.92	9.44	10.73	9.79
EX-9	8.89	14.06	13.95	12.46	6.61	9.01	10.23	9.35
EX-10	7.22	11.50	11.33	10.23	5.20	7.11	8.14	7.49

**Table 3:** Minimum buckling stress in laminated plates (EX-1 through EX-10, see Table 1) under complex in-plane loading for Cases 5–6 (Variant I—complete stiffness matrix ABD).

Laminate	*l/b =* 1		*l/b =* 1.5	
	Case 5	Case 6	Case 5	Case 6
EX-1	81.02	22.04	64.47	15.42
EX-2	108.16	28.37	88.42	20.55
EX-3	30.92	7.63	25.10	6.02
EX-4	108.68	27.19	89.56	20.90
EX-5	73.16	19.71	59.69	14.02
EX-6	75.23	20.88	61.98	14.86
EX-7	81.54	21.29	64.85	16.45
EX-8	103.21	27.02	81.48	20.80
EX-9	100.04	26.23	78.71	20.13
EX-10	81.01	21.84	65.06	15.71

**Table 4:** Dimensionless reduction coefficients α_1_, α_2_, and α_6_ in Equation (12) for the tested laminates.

Laminate	$\alpha_{1}$	$\alpha_{2}$	$\alpha_{6}$
EX-1	0.837	0.876	0.843
EX-2	0.998	0.999	0.999
EX-3	0.262	0.633	1.0
EX-4	1.0	1.0	1.0
EX-5	0.961	0.961	1.0
EX-6	0.987	0.987	1.0
EX-7	0.980	0.987	0.865
EX-8	1.0	1.0	1.0
EX-9	0.972	0.972	1.0
EX-10	0.885	0.981	0.459

## Data Availability

The original contributions presented in this study are included in the article. Further inquiries can be directed to the corresponding author.

## Appendix A

This study uses the classical laminated plate theory (CLPT) [14,15,16,17]. It was assumed that the composite materials used to fabricate the plates were subject to Hooke’s law. The plates were subjected to complex in-plane loading. It was assumed that the normal cross-sectional loads *N*_x_ and *N*_y_ were linearly variable in the direction of the 0*x*- and 0*y*-axes, whereas the shear load components *Nxy* would vary parabolically. Such a distribution of the components has been reported in the literature [14,15,16,20,21,22] for isotropic materials. The shear stress distributions are described by a relation proposed by Zhuravskii, which is a well-known formula in the literature [22,23,24]. In the thin-walled plate theory [14,15,16,17,21], it is taken that the components of the cross-sectional loads, denoted by *N**x*, *N**y*, *N**x**y*, are expressed in relation to plate edge length (and expressed in N/mm). Therefore, the stress state components correspond to the cross-sectional loads: *N*_x_, *N*_y_, *N*_xy_ divided by plate thickness.

For each of the plates, the following geometrical assumptions were considered:

(A1) $$\varepsilon_{x}=u_{,x}+0.5w_{,x}^{2},\;\varepsilon_{y}=v_{,y}+0.5w_{,y}^{2},\;2\varepsilon_{xy}=\gamma_{xy}=u_{,y}+v_{,x}+w_{,x}w_{,y},$$

and

(A2) $$\kappa_{x}=-w_{,xx},\;\;\;\;\;\;\;\;\;\;\;\;\kappa_{y}=-w_{,yy},\;\;\;\;\;\;\;\;\;\;\;\;\kappa_{xy}=-2w_{,xy},$$

where *y*, *v*, *w*—displacements in the *x, y*, and *z* directions. A reduced expression (…)_,x_ = ∂(…)/∂x, (…)_,y_ = ∂(…)/∂y has been used. According to the classical laminated plate theory (CLPT), constitutive equations for a plate have the following form [14,15,16,17]:

(A3) $$\left\{\begin{array}{c} \left\{\;N\;\right\}\\ \left\{\;M\;\right\} \end{array}\right\}=\left[\begin{array}{cc} \left[A\right] & \left[B\right]\\ \left[B\right] & \left[D\right] \end{array}\right]\left\{\begin{array}{c} \left\{\varepsilon \right\}\\ \left\{\kappa \right\} \end{array}\right\},$$

where the stiffness matrix is

(A4) $$\left[\begin{array}{cc} \left[A\right] & \left[B\right]\\ \left[B\right] & \left[D\right] \end{array}\right]=\left[\begin{array}{c} \begin{array}{cccccc} A_{11} & A_{12} & A_{16} & B_{11} & B_{12} & B_{16}\\ A_{21} & A_{22} & A_{26} & B_{21} & B_{22} & B_{26}\\ A_{61} & A_{62} & A_{66} & B_{61} & B_{62} & B_{66}\\ B_{11} & B_{12} & B_{16} & D_{11} & D_{12} & D_{16}\\ B_{21} & B_{22} & B_{26} & D_{21} & D_{22} & D_{26} \end{array}\\ \begin{array}{cccccc} B_{61} & B_{62} & B_{66} & D_{61} & D_{62} & D_{66} \end{array} \end{array}\right],$$

The A-submatrix is called tensile stiffness, the D-submatrix is known as flexural stiffness, and the B-submatrix is called a coupling (or interaction) stiffness submatrix. The submatrices [A], [B], and [D] are symmetric.

Variational methods derived differential equilibrium equations for a deformed plate element. After grouping terms containing the same variations and setting each variation in the group to zero (due to the mutual independence of the variations), the following equations were obtained:

(A5) $$\int_{S}\left[N_{x,x}+N_{xy,y}\right]\delta udS=\int_{S}X_{1}\delta udS=0,\;\int_{S}\left[N_{xy,x}+N_{y,y}\right]\delta vdS=\int_{S}X_{2}\delta vdS=0,\;\int_{S}\left[M_{x,x}+2M_{xy,xy}+M_{y,y}+N_{x}w_{,x}+{2N}_{x}w_{,xy}+N_{y}w_{,y}\right]\delta wdS=\int_{S}X_{3}\delta wdS=0,$$

- for *x =* 0; l

(A6)
$$\int_{0}^{b}\left[N_{x}-N_{x}^{0}\right]\delta udy=0,\;\int_{0}^{b}\left[N_{xy}-N_{xy}^{0}\right]\delta vdy=0,\;\int_{0}^{b}M_{x}\delta w_{,x}dy=0,\;\int_{0}^{b}\left[M_{x,x}+2M_{xy,y}+N_{x}w_{,x}+N_{xy}w_{,y}\right]\delta wdy=0,$$

- for *y =* 0; *b*

(A7) $$\int_{0}^{l}\left[N_{y}-N_{y}^{0}\right]\delta vdx=0,\;\int_{0}^{l}\left[N_{xy}-N_{xy}^{0}\right]\delta udx=0,\;\int_{0}^{l}M_{y}\delta w_{,y}dx=0,\;\int_{0}^{l}\left[M_{y,yx}+2M_{xy,x}+N_{y}w_{,y}+N_{xy}w_{,x}\right]\delta wdx=0,$$

and for the corner for *x =* const and *y =* const:

(A8) $$2M_{xy}\delta w=0,$$

where $N_{x}^{0},N_{y}^{0},\;N_{xy}^{0}$—pre-buckling external load in the plate middle surface; *dS = dl*db* has been employed in the above relation.

From Equation (A5), follow the differential equations of equilibrium for the multi-ply composite plate:

(A9) $$N_{x,x}+N_{xy,y}=0,\;N_{xy,x}+N_{y,y}=0,\;M_{x,x}+2M_{xy,xy}+M_{y,y}+{\left(N_{x}w_{,x}\right)}_{,x}{+\left(N_{y}w_{,y}\right)}_{,y}+{\left(N_{xy}w_{,x}\right)}_{,y}+{\left(N_{xy}w_{,y}\right)}_{,x}=0.$$

Considering the first two equations in Equation (A9), the third equation takes the following form:

(A10) $$M_{x,x}+2M_{xy,xy}+M_{y,y}+N_{x}w_{,x}+{2N}_{x}w_{,xy}+N_{y}w_{,y}=0.$$
